# Physicians' Attitude and Perception Towards Social Media Medical Consultation

**DOI:** 10.7759/cureus.33671

**Published:** 2023-01-11

**Authors:** Sahal J Samarkandy, Saad Samargandy, Renad A Abbas, Abdullah Alshareef, Najla Nassar, Aseel Alharbi

**Affiliations:** 1 Dermatology, Ministry of National Guard Health Affairs, Jeddah, SAU; 2 Dermatology, King Abdullah International Medical Research Center, Jeddah, SAU; 3 College of Medicine, King Saud Bin Abdulaziz University for Health Sciences, Jeddah, SAU; 4 Community Medicine, King Abdulaziz University, Jeddah, SAU; 5 Medicine, Security Forces Hospital, Makkah, SAU; 6 Dermatology, King Fahad Central Hospital, Jizan, SAU

**Keywords:** electronic questionnaire, virtual consultation, e-medicine, telemedicine, social media consultation

## Abstract

Aim: Social media plays a major role in modern healthcare. However, little is known about physicians’ experience as related to medical consultation through social media, such as Twitter. This study aims to characterize physicians’ attitudes and perceptions toward medical consultations through social media and estimate the use of social media for medical consultation purposes.

Materials and methods: The study was conducted through the distribution of electronic questionnaires to physicians from different specialities. A total of 242 healthcare providers responded to the questionnaire.

Results: Our results demonstrated that 79% of the healthcare providers received consultations through social media at least “sometimes” and that 56% of them agreed that it is appropriate to have personal social media platforms that can be accessible by patients. They also agreed (87%) that it is appropriate to interact with patients on social media; however, most of them do not find social media platforms appropriate for diagnosis or treatment.

Conclusion: Physicians think positively of social media consults, but do not consider it a proper method to manage medical conditions.

## Introduction

Social media plays an important role in modern civilized life [1]. It is easily considered a revolution in human communication that gave people an optimal opportunity to use platforms as vital resources for public information [2]. Twitter, as a platform, is considered the most common social media application used for research purposes in scientific studies by most researchers. In addition, the use of social media for promotion, which is an inevitable strategy for the development of marketing strategies, has been primarily incorporated in the medical field by health experts and medical facilities [3].

Researchers state that 33% of plastic surgeons who incorporate social media into their medical practice have noticed a positive change in the doctor-patient relationship due to the cost-effectiveness and faster approach to communication provided by social media [4]. Through social media platforms, such as Twitter, Whatsapp, and Facebook, patients can have a direct way to contact their doctors, take professional advice instantly, and avoid taking unfiltered advice from friends and family members [5]. Additionally, social support can be easily given through social media platforms by forming special support groups or pages where patients can share their experiences and support each other [5]. In another paper, researchers stated that 95% of patients read about their medical conditions through the internet before visiting physicians or taking their consultations [6]. In addition, it was stated that 45% of plastic surgeons believe that social media has made their consultations easier, while 29% of them disagree and consider this approach more difficult than standard consultations.

Other than being time-consuming, the two main reasons physicians avoid using social media are due to concerns regarding professionalism and patient confidentiality. Most literature emphasized patients’ perspectives and behaviors toward social media regarding various medical aspects but has not given enough emphasis on physicians’ perspectives, most of the time. Social media has enabled users to connect well beyond physical boundaries, drawn by geography, institution, and community [7]. Thus, this study aims to estimate the use of social media for medical consultation purposes in Saudi Arabia and characterize physicians’ perceptions and attitudes towards medical consultations through social media.

## Materials and methods

This study is a cross-sectional observational study that was carried out in multiple local tertiary hospitals in Saudi Arabia. The research subjects include 242 medical physicians from different specialties working at the intended hospitals. The Institutional Review Board of King Abdullah International Medical Research Center (KAIMRC) issued approval for this study (Approval no: IRBC/0303/21).

In this study, consultants, specialists, registrar, senior registrar, and board-certified doctors were considered. Data was collected via the distribution of online electronic questionnaires. All data are kept private with the principal investigator and were only handled by the research team. The questionnaire is based on a survey used in a previous research study from Australia [8]. The categories discussed in the questionnaire are demographics, current online usage, general online behavior, online personal information, online patient information, and appropriateness of patient-doctor online interactions. “Online” means all social media platforms which allow for social interactions or telecommunication with a widespread base of users.

For the data analysis, we calculated proportions for categorical variables and means/medians for numerical variables. T-test was used to compare the means of continuous variables, and the chi-square test was used to compare the frequencies of categorical variables. The level of significance was set at 0.05. Linear regression analysis was also used to examine risk factors. SPSS version 26 (IBM Corp., Armonk, NY) was used for statistical analysis.

## Results

A total of 242 health care providers responded to the questionnaire, most of whom were males (72.3%). Respondents were of various specialties including pediatrics (14.5%), internal medicine (10.3%), family medicine (8.7%), general surgery (8.7%), orthopedic surgery (4.5%), and several other specialties, as shown in Table 1. It was reported by the health care providers that they receive consultations through social media “all the time” (13.2%), “most of the time” (22.7%), “sometimes” (42.6%), “rarely” (9.5%), and “never” (12.0%). A large number of physicians reported that they do interact with social media consultations when they receive them (87.2%). It was reported that the most commonly used social media platforms were WhatsApp (69.4%) followed by Twitter (55.0%) (Table 2).

**Table 1:** General description of study participants

Table 1. General description of study participants	
Variables	Total participants
	(n=242)
Median Age (IQR)	40 (11)
Gender (%)	
Female	67 (27.7)
Male	175 (72.3)
Specialties, N (%)	
Pediatrics	35 (14.5)
Internal Medicine	25 (10.3)
Family Medicine	21 (8.7)
Gastroenterology	21 (8.7)
Obstetrics and Gynecology	18 (7.4)
Dermatology	13 (5.4)
Orthopedic Surgery	11 (4.5)
Psychiatry	9 (3.7)
Emergency Medicine	8 (3.3)
Other specialties	81 (33.5)
Years since promotion to consultant position by SCFHS, N (%)	
<1 year	22 (9.1)
2- <5 years	77 (31.8)
5-10 years	60 (24.8)
>10 years	74 (30.6)
Type of practice, N (%)	
Private	19 (7.9)
Public	146 (60.3)
Both private and public	77 (31.8)

**Table 2:** Social media platforms usage *Not commutative

Table 2. Social media platforms usage	
Variables	Total participants
	(n=242)
Do you receive medical consultation through social media? N (%)	
All the time	32 (13.2)
Most of the time	55 (22.7)
Sometimes	103 (42.6)
Rarely	23 (9.5)
Never	29 (12.0)
Interaction with patients, N (%)	
No	31 (12.8)
Yes	211 (87.2)
Social Media Platforms used, N (%)*	
YouTube	2 (0.8)
Facebook	7 (2.9)
Telegram	9 (3.7)
WhatsApp	168 (69.4)
Email	43 (17.8)
Twitter	133 (55.0)
Snapchat	35 (14.5)
Instagram	19 (7.9)
SMS	39 (16.1)

A Likert scale is a psychometric scale commonly involved in research that employs questionnaires. Using a Likert scale, when asked if physicians thought it was appropriate professionally to interact with patients through social media, 38.0% of them agreed that it was. However, when asked if using social media consultations was an appropriate way to seek a diagnosis and treatment, most of them disagreed. 28.1% found seeking diagnosis inappropriate whereas 33.5% thought seeking treatment was inappropriate. Health care providers do not consider it appropriate to look up a patient’s profile on social media (35.1%); however, they render it appropriate for a doctor to maintain a personal public social media profile that can be accessed by patients (39.7%) (Table 3).

**Table 3:** Physician opinions regarding social media use * Ranges from 1-5 with higher values indicating more disagreeing and 3 being neutral.

Table 3. Physician opinions regarding social media use		
Variables	Total participants	
	(n=242)	
	N (%)	Likert-scale *
		Mean (±SD)
Do you think it is appropriate for a doctor to interact with his or her patients professionally through social media?		2.68 (±1.09)
Strongly agree	27 (11.2)	
Agree	92 (38.0)	
Neutral	76 (31.4)	
Disagree	26 (10.7)	
Strongly disagree	21 (8.7)	
Do you think that social media use for medical consultation seeking diagnosis is an appropriate way of medical consultation?			3.52 (±1.14)
Strongly agree	9 (3.7)		
Agree	41 (16.9)		
Neutral	66 (27.3)		
Disagree	68 (28.1)		
Strongly disagree	58 (24.0)		
Do you think that social media use for medical consultation seeking treatment is an appropriate way of medical consultation?		3.46 (±1.05)	
Strongly agree	4 (1.7)	
Agree	47 (19.4)	
Neutral	67 (27.7)	
Disagree	81 (33.5)	
Strongly disagree	43 (17.8)	
Do you think it is appropriate for a doctor to maintain a personal public social media account that could be found by patients?			2.47 (±1.02)
Strongly agree	40 (16.5)		
Agree	96 (39.7)		
Neutral	68 (28.1)		
Disagree	29 (12.0)		
Strongly disagree	9 (3.7)		
Do you think it is appropriate for doctors to look up publicly available online information about a patient in an emergency? e.g., searching a patient’s Twitter account for information following a suicide attempt.		3.76 (±1.15)	
Strongly agree	8 (3.3)	
Agree	27 (11.2)	
Neutral	65 (26.9)	
Disagree	57 (23.6)	
Strongly disagree	85 (35.1)	

**Figure 1:**
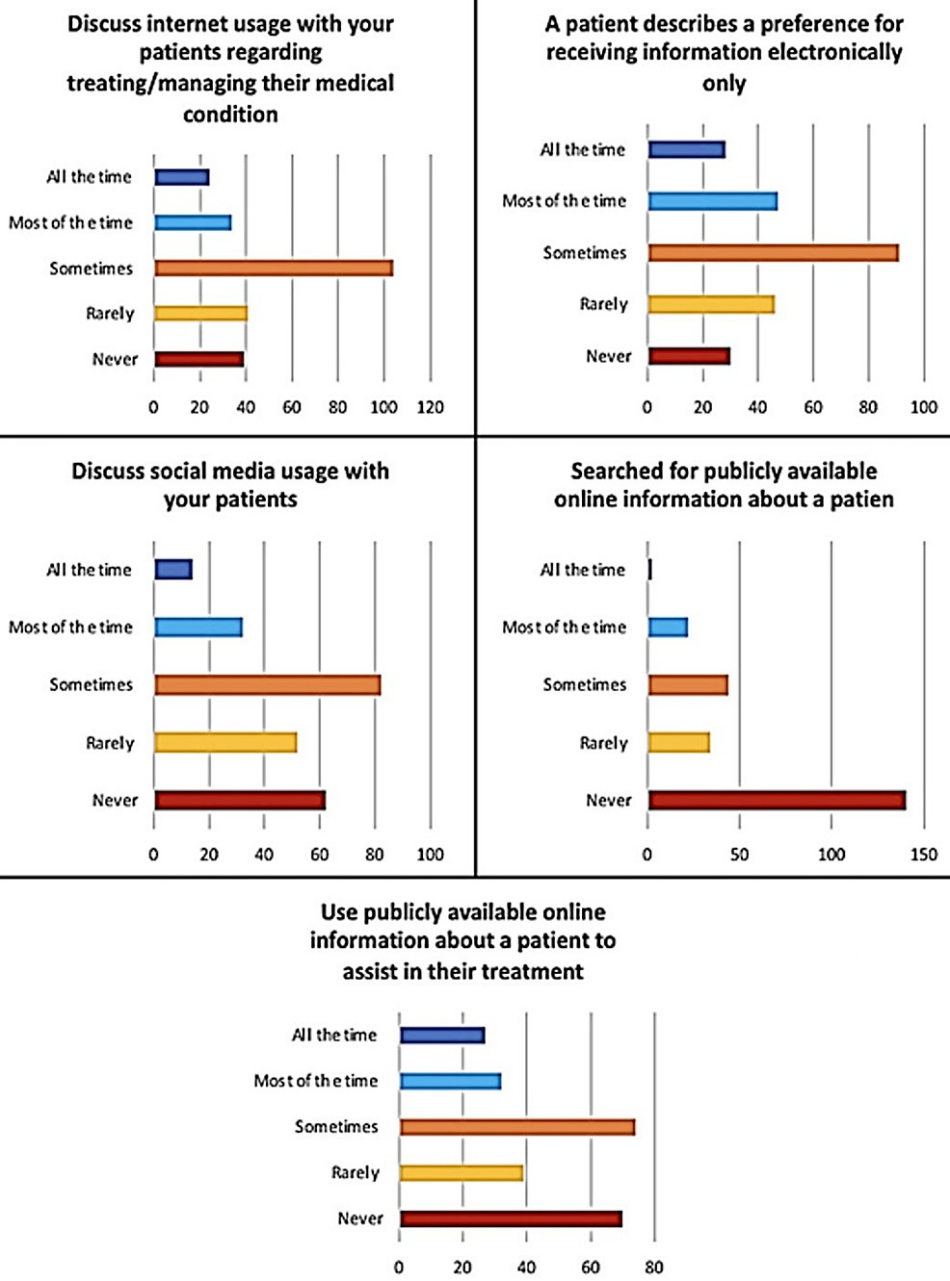
Frequency of actions taken by physicians regarding social media usage

This study questioned if health care providers discussed internet usage with their patients as an aid for treating or managing their medical conditions, for example, helping patients access online information about their disease, to which most of them answered “sometimes”. They were also asked if they would cooperate if their patient’s expressed a preference for receiving information electronically, to which they also answered they “sometimes” would. Another question asked to the health providers was if they were to use publicly available online information about a patient in order to assist the patient's medical management, most of them also answered “sometimes”. Another question inquired if health care providers discussed social media usage with their patients, such as online support groups for certain medical conditions to which they responded that they “rarely” do. Health care providers were also asked if at any time they searched for publicly available online information about a patient, for example, used Google to find more information about their patients, to which most of them answered “never” (Figure 1).

**Figure 2:**
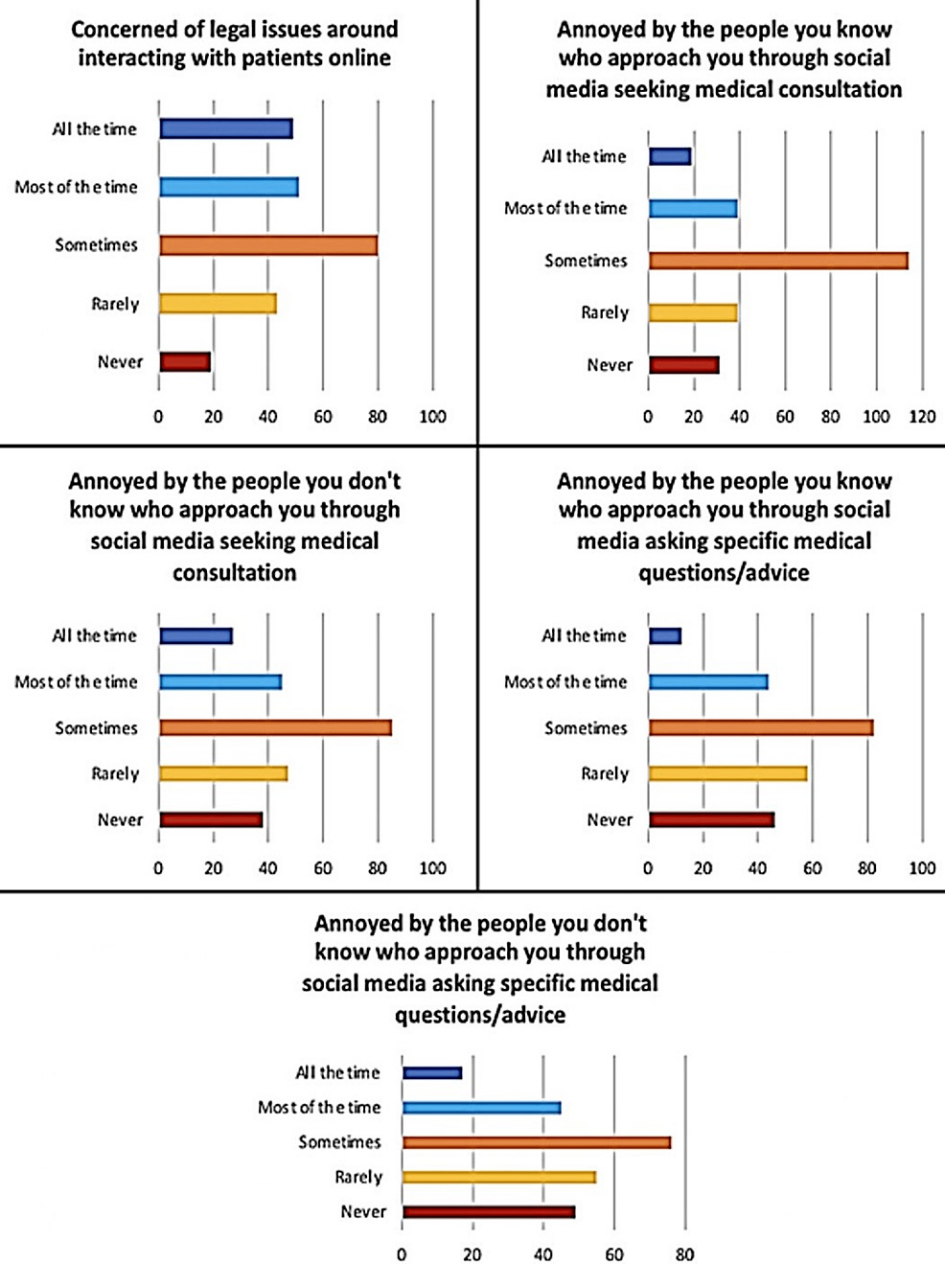
Frequency of concerns of physicians regarding social media usage

For the final part of the questionnaire, health care providers were assessed regarding concerns. To exemplify, when they were asked if they were concerned about legal issues regarding interacting with patients online, most of them answered “sometimes”. They were also asked if they were annoyed with people they know (friends and family) approaching them on social media, most of them responded with “sometimes”. In addition, when asked if they were annoyed with people they do not know approaching them on social media, most of them answered “sometimes”. When asked again if they are annoyed with both strangers and acquaintances approaching them on social media seeking certain medical answers about certain conditions, most of them similarly answered “sometimes” (Figure 2).

## Discussion

Most previous studies focused on the patients' perspectives of consultation using social media, while this research aimed to assess that from the physicians’ points of view on social media consultation. Our results demonstrate that the most used social media platform in consultations is WhatsApp, unlike a study conducted in 2013 which stated that Twitter is the number one social media platform used in consultations [1]. Although other studies [2] found that the dominating specialty in social media consultations was plastic surgery (33%), our analysis displays pediatrics as the dominating field when it comes to social media consultations (14.5%). 39.7% of physicians found it appropriate to have a public personal social media account that patients can easily find, which could be indicated as a type of promotion for their practice, whether public or private. Similarly, this is demonstrated in other studies [4] as another use for social media platforms.

Although the main limitation of social media consultations previously reported were concerns about professionalism and confidentiality, our results demonstrate otherwise. It was reported by 33.9% of our sample that it is professionally appropriate to interact with patients through social media platforms, while 11.2% disagree. In a similar study, one of the concerns of social media interactions was a depiction of unprofessional behavior. Awareness of social media use is evident across multiple disciplines, as are concerns regarding the potential for misuse [7]. The main concern reported in our study was the annoyance perceived by physicians when being approached on different social media platforms. 

The limitation of the study was that it was conducted through an online questionnaire; therefore, it was difficult to reach as many physicians as possible, targeting physicians who were only available online.

## Conclusions

The use of social media platforms for consultation and promotion is increasing daily in the medical field and is being used in all specialties. Many physicians agree that it is suitable to use social media for consultation. Not all physicians are comfortable when approached on social media by strangers, friends, or even family. Some get disturbed whether they are approached with general or specific concerns. Mostly, physicians think positively of social media consults, but do not consider it a convenient method to seek diagnosis or treatment.
